# Another Apology

**Published:** 1886-09

**Authors:** 


### ANOTHER APOLOGY.

Our excuses to contributors are becoming somewhat monotonous. But they are a necessity, for it is positively impossible to print in each number all the acceptable matter that is offered. Articles from some of the best writers in the profession are waiting their turn, and sometimes the authors think they are obliged to wait altogether too long. Yet this number consists of *seventy-six pages.* Some of the publishers have put their hands deeply into their own pockets that additional space may be given for matter that they deem of importance to the profession. How many would do the like, when they had no personal object to serve? The printing of the report of the Herbst clinics, for instance, is directly paid for by those whose only object is to give to the profession information that shall be of service to every dentist. All honor to the men who stand ready to prove their devotion to dentistry by the most unselfish of labors, and by an unstinted liberality.

